# Concurrent Resistance to Carbapenem and Colistin Among *Enterobacteriaceae* Recovered From Human and Animal Sources in Nigeria Is Associated With Multiple Genetic Mechanisms

**DOI:** 10.3389/fmicb.2021.740348

**Published:** 2021-10-06

**Authors:** Emmanuel O. Ngbede, Folasade Adekanmbi, Anil Poudel, Anwar Kalalah, Patrick Kelly, Yi Yang, Andrew M. Adamu, Salem T. Daniel, Alex A. Adikwu, Chinedu A. Akwuobu, Paul O. Abba, Levi M. Mamfe, Nanven A. Maurice, Mohammed I. Adah, Olivia Lockyear, Patrick Butaye, Chengming Wang

**Affiliations:** ^1^Department of Veterinary Microbiology, College of Veterinary Medicine, Federal University of Agriculture Makurdi, Makurdi, Nigeria; ^2^Department of Pathobiology, Auburn University College of Veterinary Medicine, Auburn, AL, United States; ^3^Department of Biomedical Sciences, Ross University School of Veterinary Medicine, Basseterre, Saint Kitts and Nevis; ^4^Yangzhou University College of Veterinary Medicine, Yangzhou, China; ^5^Department of Veterinary Public Health and Preventive Medicine, Faculty of Veterinary Medicine, University of Abuja, Abuja, Nigeria; ^6^Department of Microbiology, College of Sciences, Federal University of Agriculture Makurdi, Makurdi, Nigeria; ^7^Department of Veterinary Public Health and Preventive Medicine, College of Veterinary Medicine, Federal University of Agriculture Makurdi, Makurdi, Nigeria; ^8^Department of Medical Microbiology and Parasitology, Benue State University Teaching Hospital, Makurdi, Nigeria; ^9^Department of Diagnostics and Extension, National Veterinary Research Institute, Vom, Nigeria; ^10^Department of Veterinary Medicine, College of Veterinary Medicine, Federal University of Agriculture Makurdi, Makurdi, Nigeria; ^11^Department of Pathology, Bacteriology and Avian Diseases, Faculty of Veterinary Medicine, Ghent University, Merelbeke, Belgium

**Keywords:** concurrent carbapenem-colistin resistance, *Enterobacteriaceae*, high-risk clones, Nigeria, Africa, whole genome sequencing

## Abstract

Resistance to last resort drugs such as carbapenem and colistin is a serious global health threat. This study investigated carbapenem and colistin resistance in 583 non-duplicate *Enterobacteriaceae* isolates utilizing phenotypic methods and whole genome sequencing (WGS). Of the 583 isolates recovered from humans, animals and the environment in Nigeria, 18.9% (110/583) were resistant to at least one carbapenem (meropenem, ertapenem, and imipenem) and 9.1% (53/583) exhibited concurrent carbapenem-colistin resistance. The minimum inhibitory concentrations of carbapenem and colistin were 2–32 μg/mL and 8 to >64 μg/mL, respectively. No carbapenem resistant isolates produced carbapenemase nor harbored any known carbapenemase producing genes. WGS supported that concurrent carbapenem-colistin resistance was mediated by novel and previously described alterations in chromosomal efflux regulatory genes, particularly *mgrB* (M1V) *ompC* (M1_V24del) *ompK37* (I70M, I128M) *ramR* (M1V), and *marR* (M1V). In addition, alterations/mutations were detected in the *etpA, arnT, ccrB, pmrB* in colistin resistant bacteria and *ompK36* in carbapenem resistant bacteria. The bacterial isolates were distributed into 37 sequence types and characterized by the presence of internationally recognized high-risk clones. The results indicate that humans and animals in Nigeria may serve as reservoirs and vehicles for the global spread of the isolates. Further studies on antimicrobial resistance in African countries are warranted.

## Introduction

Antimicrobial resistance, particularly in Gram-negative bacteria, challenges the ability to treat common infections and is one of the greatest threats to global public health systems (Breijyeh et al., 2020). Resistance is more worrisome in resource-limited countries such as in sub-Saharan Africa where infections are common and last-resort antimicrobial agents are scarce and/or unaffordable (Tompkins et al., 2021).

Carbapenems and colistin play a significant role as “last resort” antibiotics in the treatment of infections caused by an extended spectrum of β-lactamase producing *Enterobacteriaceae* and multidrug-resistant *Enterobacteriaceae* (including carbapenem-resistant isolates), respectively. Categorized as the highest priority critically important drugs, the emergence of resistance to them, both individually and concurrently, is a serious source of healthcare concern (World Health Organization [WHO], 2018). Globally, resistance to colistin and carbapenem has been increasingly reported in isolates from animals and humans. The concurrent resistance of *Enterobacteriaceae* to carbapenems and colistin has been reported with increasing frequency in some parts of the world (Du et al., 2016; Yao et al., 2016; Lomonaco et al., 2018). Concurrent resistance determinants are usually located on conjugative plasmids and can thereby be co-transferred, setting the stage for pandrug resistance (Long et al., 2019).

Carbapenem resistant *Enterobacteriaceae* (CRE) comprise both carbapenemase producing (CP-CRE) and non-carbapenemase producing CRE (non-CP-CRE) strains. While CPE produce carbapenemases to hydrolyze carbapenem, non-CP-CRE have β-lactamase (ESBLs and AmpC enzymes) activity combined with structural mutations of the outer membrane protein and drug efflux pumps (Logan and Weinstein, 2017).

Colistin resistance in *Enterobacteriaceae* can be due to structural modifications of the bacterial lipopolysaccharide, such as the addition of 4-amino-4-deoxy-l-arabinose (L-Ara4N) or phosphoethanolamine (pEtN) (intrinsic resistance). Acquired resistance may result from chromosomal mutations in genes encoding the *PhoPQ* and *PmrAB* a two-component regulatory system, the *mgrB*, a negative regulator of *PhoPQ*, or the plasmid-borne mobile colistin resistance genes (*mcr*-1 to *mcr*-10) encoding a group of pEtN transferases (Aires et al., 2016; Zafer et al., 2019; Wang C. et al., 2020).

We recently reported the occurrence of *mcr*-mediated colistin resistance among 99 colistin resistant isolates; *Escherichia coli* (67/99), *Klebsiella pneumoniae* (30/99), *Citrobacter werkmanii* (1/99), and *Alcaligenes faecalis* (1/99) from humans and animals in Nigeria (Ngbede et al., 2020). Studies have also reported a high prevalence, up to 52%, of carbapenem resistance mediated by carbapenemase producing genes in samples from humans in Nigeria (Ogbolu and Webber, 2014; Jesumirhewe et al., 2017; Olowo-okere et al., 2019; Otokunefor et al., 2019; Ogbolu et al., 2020; Olalekan et al., 2020; Olowo-Okere et al., 2020; Shettima et al., 2020). These findings suggest that resistance to the last resort drugs carbapenem and colistin is a significant problem in Nigeria. Despite these increasing reports on colistin and carbapenem resistant bacteria emanating from sub Saharan Africa, the occurrence of co-resistance to both drugs and detailed insight into the molecular mechanism associated with this resistance phenotype has not been a major focus of such studies. Similarly, majority of these studies focus mainly on PCR detection of the most commonly and previously reported mechanisms including those mediated by *bla_*KPC*_, bla_*NDM*_, bla_*IMP*_* genes for carbapenem and the *mcr*- gene for colistin and rarely followed by WGS analyses of the isolates which is usually targeted at strain typing. This is in spite of the increasing non-detection of these genes in some of the isolates resistant to these antibiotics. Understanding the mechanisms of resistance is crucial to countering the mounting burden of infections caused by multidrug resistant bacteria, and whole-genome sequencing (WGS) has been shown to play a significant role in the rapid and accurate detection and characterization of known and emerging resistance determinants. Rarely, studies from sub Saharan Africa utilized whole genome sequencing to understand the detailed underlying genetic mechanism for carbapenem-colistin co-resistance in *Enterobacteriaceae* to the best of our knowledge. Such information is critical in formulating strategies for the clinical management and control of infections caused by multidrug-resistant *Enterobacteriaceae*. This study aimed to investigate the prevalence and genetic mechanisms underlying colistin-carbapenem co-resistance among *E. coli* and *Klebsiella* species (two important nosocomial pathogens) recovered from animals, humans, and the environment in Nigeria.

## Materials and Methods

### Bacteria Isolates

This study utilized a total of 583 non-duplicate *Enterobacteriaceae* acquired between 2016 and 2019. Isolates comprised 487 *E. coli*, 87 *Klebsiella* species, and nine *Citrobacter* species and were recovered from human clinical sample: stool (*n* = 60) and urine (*n* = 35), human hospital environment (*n* = 15), rectal swabs of camels (*n* = 40), cattle (*n* = 36), dogs (*n* = 42), pigs (*n* = 65), and cloacal swabs of poultry (*n* = 250) and clinical samples (liver) of poultry (*n* = 40) were used in this study (Ngbede et al., 2020). Procedures for sample collection, isolation, and identification have been previously reported (Ngbede et al., 2020). Ethical approval for the collection of samples from humans was provided by the Health Research and Ethics Committee of the facilities (FMH/FMC/MED.108/VOL.I/X and BSUTH/MKD/HREC/2013B/2018/0027).

### Determination of Minimum Inhibitory Concentration

Minimum inhibitory concentrations (MICs) of carbapenem (meropenem, ertapenem, and imipenem) were determined on the 583 isolates using the broth dilution method as recommended by the Clinical and Laboratory Standards Institute (CLSI) (Clinical and Laboratory Standards Institute, 2020). Briefly, colonies of each isolate from an overnight growth on tryptone soya agar were suspended in 5 mL normal saline to make an inoculum the equivalent of a 0.5 MacFarland standard. The turbidity of the inoculum was measured using a densitometer (BioScan). A 20 μL volume of the inoculum was dispensed into 2 mL of Mueller–Hinton broth containing different concentrations of the respective carbapenems and incubated at 35°C for 24 h. *E. coli* ATCC 25922 and *Pseudomonas aeruginosa* ATCC 27853 served as controls.

### Phenotypic Assay for Carbapenemase Production

The Carbapenem inactivation method (CIM) was used to screen the isolates for carbapenemase production as described (van der Zwaluw et al., 2015). Briefly, a loopful (≈10 μL) of the isolate was suspended in 400 μL of double distilled water and followed by a 10 μg meropenem disk (Oxoid, United Kingdom) which was immersed in the suspension and incubated for 2 h at 35°C. The disk was removed from the suspension with an inoculation loop and placed on a Mueller–Hinton agar plate inoculated with a 0.5 McFarland standard *E. coli* strain ATCC 29522 (a susceptible indicator strain) using a sterile cotton swab; it was subsequently incubated at 35°C for 24 h. A positive results was provided when isolates with carbapenemase production inactivates meropenem in the disk, allowing uninhibited growth of the susceptible indicator strain. A negative result occurred when meropenem disks were incubated in suspensions of isolates without carbapenemases, yielding a clear inhibition zone of the susceptible indicator strain (van der Zwaluw et al., 2015).

### Antimicrobial Susceptibility Testing

Antimicrobial susceptibility profiles of the isolates were determined by the disk diffusion method (Clinical and Laboratory Standards Institute, 2020) using 10 antimicrobial agents sourced from Oxoid, United Kingdom: Amoxycillin (10 μg), amoxicillin/clavulanic acid (30 μg), cefoxitin (30 μg), ceftriaxone (30 μg), ciprofloxacin (5 μg), chloramphenicol (10 μg), gentamicin (10 μg), doxycycline (30 μg), enrofloxacin (5 μg), and sulfamethoxazole-trimethoprim (25 μg). The results were interpreted based on the guidelines of the CLSI (Clinical and Laboratory Standards Institute, 2020).

### DNA Extraction and Whole Genome Sequencing

Fifty out of the 53 isolates co-resistant to colistin and carbapenem, were randomly selected and subjected to WGS. Only 50 isolates were randomly selected for the WGS due to its associated cost and the selection of isolates represented the different species and susceptibility patterns encountered among the 53 co-resistant (carbapenem-colistin) isolates recovered from humans, animals and the environment. Genomic DNA (gDNA) was extracted from overnight cultures of the isolates using the Wizard Genomic DNA Purification Kit (Promega, United States) following the manufacturer’s recommendations. Isolates were sequenced using next-generation sequencing on an Illumina MiSeq platform (OE Biotech, Shanghai, China) using the V2 paired-end chemistry (2 × 250 bp).

### Analyses of WGS Data

Quality of the sequencing was assessed using QUAST v5.1^1^ (Gurevich et al., 2013) before *de novo* assembly using the SeqMan Pro v.11.2.1 (DNASTAR, United States) followed by annotation using PROKKA v1.14.5. Species identity of the isolates was further confirmed with the WGS data using the KmerFinder v3.2^2^ (Hasman et al., 2014; Larsen et al., 2014; Clausen et al., 2018). Genetic relatedness of isolates was analyzed by phylogroup (*E. coli* only), multilocus sequence types (MLST) and core genome MLST (cgMLST). MLST for each isolate was predicted using the pubMLST^3^ while the assignment of core genome MLST and phylogroups for the *E. coli* isolates were carried out using the cgMLSTFinder v1.1^4^ (Zhou et al., 2020) and the online Clermont tool v20.03^5^ (Beghain et al., 2018; Clermont et al., 2019).

*In silico* serotyping for the *E. coli* and *Klebsiella* isolates was performed using the SeroFinder 2.0^6^ (Joensen et al., 2015) and Kaptive^7^ (Wick et al., 2018).

The presence of acquired antimicrobial resistance genes were investigated using the ResFinder v4.0^8^ (Bortolaia et al., 2020) and CARD RGI 5.2.0^9^ (Alcock et al., 2020). Plasmids were identified using PlasmidFinder 2.1^10^ (Carattoli et al., 2014) and MGEFinder v1.0.3^11^ (Johansson et al., 2021). Virulence genes were identified using the VirulenceFinder 2.0 (Lui et al., 2019).

### Detection of Mutations in Genes Related to Colistin and Carbapenem Resistance

Mutations in the genes previously identified as responsible for resistance to colistin (*mgrB, prmAB, phoPQ, arnT, ccrB*) and carbapenem (*ompC, ompF, ompK/35/36/37*, *marR, acrR, ramR*) were investigated by alignment with wild type reference genomes of *E. coli* strain K-12 substrain MG1655 (NC_000913.3) and *K. pneumonia* subspecies *pneumoniae* MGH 78578 (NC_009648.1). PROVEAN v1.1.3^12^ was employed to predict the possible role/effect of observed amino acid substitutions (mutation/alteration) on protein functions i.e., colistin or carbapenem resistance (Choi and Chan, 2015). The standard PROVEAN cutoff score of ≤-2.5 and >-2.5 was used to categorize the mutation with deleterious and/or neutral effects on protein function, respectively (Choi et al., 2012; Choi and Chan, 2015).

### Transfer Experiments

The conjugative transferability of colistin and carbapenem resistance determinants was assessed using the solid mating conjugation assay with sodium azide-resistant *E. coli* J53 as the recipient (Ojo et al., 2016). Overnight cultures of the donor and recipient were mixed in a 1:4 ratio in tryptone soya broth and centrifuged at 14,000 × *g* for 1 min. The pelleted cells were resuspended in 15 μL of 0.85% NaCl, spotted onto MHA, incubated at 37°C for 20–24 h. Bacteria growing on the MHA were resuspended in 1 mL of 0.85% NaCl and a 100 μL serially diluted aliquot. Each dilution was placed on Brain Heart Infusion (BHI) agar supplemented with sodium azide (150 μL/mL) + colistin (2 μg/mL), meropenem (2 μg/mL) + sodium azide (150 μL/mL), colistin (2 μg/mL) + meropenem (2 μg/mL) + sodium azide (150 μL/mL) for the selection of transconjugant. The conjugation, or transfer, the frequency was measured based on the ratio of the observed transconjugant CFU (T) divided by the recipient CFU (T/R).

## Results

### Prevalence of Carbapenem Resistance and Concurrent Carbapenem-Colistin Resistance

Of the 583 isolates tested, 110 (18.9%) comprising 53 *E. coli* and 57 *Klebsiella* species were resistant to at least one of three carbapenems (meropenem, 18.7%; 109/583; ertapenem, 16.8%; 98/583; imipenem, 18.2%; 106/583) and originated from pigs (*n* = 14), poultry (*n* = 39), humans (*n* = 33), environment (*n* = 3), cattle (*n* = 10), camel (*n* = 1), and dogs (*n* = 10). Based on colistin resistance findings determined in a former study (Ngbede et al., 2020) and this study, 53 isolates (9.1%; 53/583) were considered resistant to both colistin and at least one of the carbapenems. Of the 53 isolates expressing concurrent carbapenem-colistin resistance, 50 were randomly selected for further investigation: 23 human isolates (16 *K. pneumoniae*, 2 *K. quasipneumoniae*, and 5 *E. coli*), one hospital environmental isolate (*K. quasipneumoniae*), and 26 animal isolates (1 *K. pneumoniae*, 24 *E. coli*, and 1 *C. werkmanii*). The MIC of carbapenems ranged between 2 and 32 μg/mL (Supplementary Table 1).

The resistance profile is shown in Table 1 for the *Citrobacter* and *E. coli* isolates and Table 2 for the *Klebsiella* isolates. The 50 carbapenem/colistin resistant strains were negative for carbapenemase production, with 98% of the isolates resistant to the β-lactam antibiotics. This particularly included amoxicillin and amoxicillin/clavulanic acid as all the isolates except one *E. coli*, were resistant to amoxycillin. Similarly, 48, 66, and 78% of the isolates were resistant to aminoglycosides, sulphamethaxazole/trimethoprim, and fluoroquinolones, respectively (Tables 1, 2).

**TABLE 1:** Phenotypic and genotypic resistance profile of *Enterobacteriaceae* of animal origin expressing concurrent carbapenem-colistin resistance from Nigeria.

ID	Phenotypic resistance profile	β-lactamases	Aminoglycosides	Fluoroquinolones	Sulphamethazoxle-Trimethoprim	Tetracycline	Phenicol
B14	AMC AMX CEF DOX GEN ENR STX	CTX-M-15, TEM-1B	*aph(3″)*-*Ib, aph(6)*-*Id, aac(3)*-*IId*	qnrS1, qepA1, gyrA^*^	*sul2, dfrA14, dfrA17*	*tetA, tetB*	–
B22	AMC AMX CEF ENR STX	CTX-M-15	*aph(3″)*-*Ib, aph(6)*-*Id*	*qnrS1*	*sul2, dfrA14*		–
C40	AMC AMX CEF ENR	CTX-M-15		*qnrS1*		*tetA*	–
E41	AMC AMX CEF STX	CTX-M-15, TEM-1B	*aph(3″)*-*Ib, aph(6)*-*Id*	*qnrS1*	*sul2, dfrA14*	*tetA*	–
L3	AMC AMX CEF DOX GEN STX	CTX-M-55	*aac(3)*-*IId*	*qnrS1*	*sul2, dfrA14*	*tetA*	
L18	AMC AMX CHL CEF DOX GEN ENR FLOR SXT	TEM-1B	*armA, aadA1, aadA2b, aac(3)*-*IIa, aph(3′)*-*Ia, aph(6)*-*Ic*,	acrR^*^, qnrB1, oqxA, oqxB, qnrB17	*sul1, sul3, dfrB4*,	ramR^*^, tetD, tetM	*cmlA1*
L6	AMC AMX CIP CEF DOX ENR STX	CTX-M-15, OXA-1	*aac(6′)*-*Ib*-*cr, aadA5*	*gyrA* ^*^	*sul1, dfrA17*	*tetA*	*CatB3*
L13	AMC AMX CIP CEF DOX ENR STX	CTX-M-15, OXA-1	*aac(6′)*-*Ib*-*cr*	*gyrA* ^*^	*sul1, dfrA17*	*tetA*	*CatB3*
L15	AMC AMX CEF ENR	CTX-M-15, OXA-1	*aac(6′)*-*Ib*-*cr*	*gyrA* ^*^	*sul1, dfrA17*	*tetA*	*CatB3*
L16	AMC AMX CEF COL DOX ENR STX	CTX-M-15, TEM-1B	*aph(3″)*-*Ib, aph(6)*-*Id*	*qnrS1*	*sul2, dfrA14*	*tetA*	
L17	AMC AMX CHL CIP CEF DOX GEN ENR STX	CTX-M-15	*aadA2, aadA1*	*gyrA* ^*^	*sul2, dfrA12*	*tetA*	*cmlA1*
L20	AMC AMX CHL CIP CEF DOX GEN ENR FLOR	CTX-M-65	*aac(3)*-*IId*	*qnrS13*		*tetA*	*floR*
L22	AMC AMX CHL CIP CEF DOX GEN ENR FLOR STX	-	*aac(3)*-*IIa, aph(3′)*-*Ia, aph(3″)*-*Ib, aadA1*	*gyrA* ^*^	*sul3, dfrA1*	*tetA*	*floR*
L23	AMC AMX CHL CEF DOX GEN ENR	TEM-1B	*aac(3)*-*Via*	gyrA^*^, qnrS1	*sul1, sul3, dfrA14*	*tetA*	
L25	AMX AMC CIP CEF ENR STX	TEM-1B	*aac(3)*-*Via*	gyrA^*^, qnrS1	*sul1, sul3, dfrA14*	*tetA*	
L26	AMC AMX CHL CEF DOX ENR FLOR STX	TEM-1B	*aph(3′)*-*Ia, aph(3″)-Ib, aadA1*	gyrA^*^, qnrS1, parC^*^	*sul3, dfrA1*	*tetA*	*floR*
L27	AMC AMX CHL CEF COL GEN ENR FLOR STX	TEM-1B	*aac(3)*-*IIa, aadA1, aph(3″)*-*Ib*	*gyrA* ^*^	*sul3, dfrA1*	*tetA*	*floR*
L28	AMC AMX CIP CEF DOX GEN ENR	TEM-1A	*aac(3)*-*IId, aac(3)*-*Via*	gyrA^*^, qnrS1	*sul1, sul3, dfrA14*	*tetA*	–
L29	AMC AMX CEF DOX GEN ENR STX	TEM-1B	*aac(3)*-*Via*	gyrA^*^, qnrS1	*sul1, sul3, dfrA14*	*tetA*	–
L31	AMC AMX CEF DOX GEN	CTX-M-55	*aph(3′)*-*Ia, aph(3″)*-*Ib, aph(6)*-*Id*	gyrA^*^, parC^*^	*sul2*	*tetB*	–
L36	AMC AMX CHL CIP CEF DOX GEN ENR STX	TEM-1B	*aac(3)*-*IId, aph(3′)*-*Ia, aph(3″)*-*Ib, aph(6)*-*Id*		*sul2, dfrA14*	*tetA*	*floR*
L38	AMC AMX CHL CEF DOX GEN ENR FOX STX	CMY-98, TEM-1	*aph(3′)*-*Ia, aph(6)*-*Id, aadA2b*	*qnrB19, qnrB34*	*sul1, dfrA12*	*tetB, tetM*	*cmlA1*
L39	AMC AMX CIP CEF DOX ENR STX	TEM-1B	*aadA5*	*qnrS1*	*sul2, dfrA17*	*tetA*	–
L40	AMC AMX CHL CIP CEF DOX GEN ENR FLOR STX	TEM-1B	*aac(3)*-*IId, aph(3′)*-*Ia, aph(3″)*-*Ib, aph(6)*-*Id*	*qnrS1*	*sul2, dfrA14*		*floR*
L41	AMC AMX CEF DOX GEN ENR		*aac(3)*-*IIa, aph(6)*-*Id*	qnrS13, gyrA^*^, parC^*^,	*sul2*	*tetA*	–
L43	AMC AMX CIP CEF GEN ENR	TEM-1B	–	*qnrS1*	–	–	–

^*^Mutations on the respective genes.

*AMC, amoxycillin; AMX, amoxycillin-clavulanic acid; CHL, chloramphenicol; CEF, ceftriaxone; CIP, ciprofloxacin; DOX, doxycycline; FLOR, florfernicol; GEN, gentamicin; ENR, enrofloxacin; FOX, cefoxitin; STX, sulfamethazaxole-trimethoprim.*

**TABLE 2:** Phenotypic and genotypic resistance profile of *Enterobacteriaceae* of human origin expressing concurrent carbapenem-colistin resistance from Nigeria.

ID	Phenotypic resistance profile	β-lactamases	Aminoglycosides	Fluoroquinolones	Sulphamethazoxle-Trimethoprim	Tetracycline	Phenicol
H2	AMC AMX CEF GEN	SHV-11	–	acrR^*^, oqxA, oqxB	–	–	–
H34	AMC AMX CHL CIP CEF DOX ENR FOR STX	CTX-M-15, TEM-1B	*aac(6′)*-*Ib*-*cr, aac(6′)*-*Ib3*	gyrA^*^, parC^*^, parE^*^	*sul1, dfrA1*	*tetB*	*catA1, catB3*
H35	AMC AMX CHL CIP CEF	CTX-M-15	*aadA1*	qepA4, gyrA^*^, parE^*^	*sul1, sul2, dfrA12*	*tetB*	*catA1, cmlA1*
H36	AMC AMX CEF DOX STX	SHV-1	–	acrR^*^, oqxA, oqxB	–	–	–
H41	CEF COL DOX	–	–	–	–	–	
H6	AMC AMX CHL CIP CEF GEN STX	TEM-1B, SHV-11	*aac(3)*-*IId, aadA1, aph(3′)*-*Ia, aph(3″)*-*Ib, aph(6)*-*Id*	acrR^*^, oqxA, oqxB, qnrS1	*sul2, dfrA14*,	*tetA, tetD*	*catA2*
H50	AMC AMX CEF DOX STX	OKP-B-8	–	acrR^*^, oqxA, oqxB	–	–	–
H4	AMC AMX CHL CIP CEF DOX ENR FOX STX	SHV-1	–	acrR^*^, oqxA, oqxB	–	–	–
H5	AMC AMX CEF DOX ENR STX	TEM-1B, DHA-1, SHV-1	*aph(3″)*-*Ib, aph(6)*-*Id*	acrR^*^, oqxA, oqxB, qnrB4	*sul1, sul2, dfrA1, dfrA14*,		
H7	AMC AMX CEF DOX ENR	SHV-11	*aadA1*	*acrR* ^*^	*sul2, dfrA5*		
H23	AMC AMX CIP CEF DOX GEN ENR SXT	CTX-M-15, OXA-1, TEM-1B, SHV-28	*aac(3)*-*IIa, aac(6′)*-*Ib*-*cr, aph(3″)*-*Ib, aph(6)*-*Id*	acrR^*^, oqxA, oqxB, qnrB1	*sul2, dfrA14*	*tetA*	
H25	AMC AMX CIP CEF DOX GEN ENR STX	CTX-M-15, OXA-1, TEM-1B, SHV-28	*aac(3)*-*IIa, aac(6′)*-*Ib*-*cr*	acrR^*^, oqxA, oqxB, qnrB1	*sul2, dfrA14*,	*tetA*	*catB3*
H26	AMC AMX CIP CEF GEN ENR STX	CTX-M-15, OXA-1, TEM-1B, SHV-28	*aac(3)*-*IIa, aac(6′)*-*Ib*-*cr*	acrR^*^, oqxA, oqxB, qnrB1	*sul2, dfrA14*,	*tetA*	*catB3*
H29	AMC AMX CEF FLOR	–	–	–	*sul2, dfrA15*		–
H30	AMC AMX CEF FLOR MER	OKP-B-7	–	acrR^*^, oqxA, oqxB	–	–	–
H31	AMC AMX CEF DOX ENR FLOR STX	SHV-1	–	acrR^*^, oqxA, oqxB	–	–	–
H39	AMC AMX CEF DOX	TEM-1C, SHV-1	*aadA1*	*oqxA, oqxB*	*sul1, dfrA1*	*tetA*	
H40	AMC AMX CEF		*aph(3′)*-*Ib, aph(6)*-*Id*	acrR^*^, oqxA, oqxB			
H45	AMC AMX CHL CIP CEF DOX GEN ENR FLOR STX	CTX-M-15, TEM-1B, SHV-11	*aac(3)*-*IId, aadA2*	acrR^*^, oqxA, oqxB	*sul1, sul2 dfrA1*	*tetD*	*catA2*
H46	AMC AMX CHL CIP CEF DOX GEN ENR FLOR STX	CTX-M-15, TEM-1B, SHV-11	*aac(3)*-*IId, aadA2, ant(2″)*-*Ia, aph(3′)*-*Ia, aph(3′)*-*Ib*	*oqxA, oqxB*	*sul1, sul2 dfrA10, dfrA12*	*tetD, tetJ*	
H47	AMC AMX CHL CIP CEF GEN STX	CTX-M-15, TEM-1B, SHV-11	*aac(3)*-*IId, aadA2, aph(3′)*-*Ib*	acrR^*^, oqxA, oqxB	*sul1, sul2 dfrA12*	*tetD*	*catA2*
H48	AMC AMX CEF ERY FOX STX	TEM-1B, SHV-11	–	acrR^*^, oqxA, oqxB	*sul2 dfrA26*	–	–
H49	AMC AMX CEF	TEM-1B, DHA-17	*aph(3″)*-*Ib, aph(6)*-*Id, aadA1*	*sul1, sul2*	*dfrA1, dfrA5*	*tetA*	*catA2*
H22	AMC AMX CEF DOX ENR STX	OKP-B-5	*aph(3″)*-*Ib, aph(6)*-*Id*	acrR^*^, oqxA, oqxB			

^*^Mutations on the respective genes.

*AMC, amoxycillin; AMX, amoxycillin-clavulanic acid; CHL, chloramphenicol; CEF, ceftriaxone; CIP, ciprofloxacin; DOX, doxycycline; FLOR, florfernicol; GEN, gentamicin; ENR, enrofloxacin; FOX, cefoxitin; STX, sulfamethazaxole-trimethoprim.*

### Whole Genome Sequence Analysis

Quality assessment of the genome reads revealed the *Citrobacter* isolate had a total genome length of 5.0 MB distributed over 60 contigs with an N_50_ and average GC content of 48,746 bp and 52, respectively. The total genome length of the *E. coli* isolates ranged from 3.7 to 4.2 MB distributed over 50–1465 contigs with an N_50_ length of 2,642–296,122 bp and an average GC content (mol%) of 50 while the *Klebsiella* isolates had an average GC content (mol%) of 50, total genome and N_50_ length of 5.0–5.6 MB and 126,035–462,504 bp, respectively distributed over 24–195 contigs. The WGS data further confirmed the identity of the 50 isolates as *C. werkmanii* (*n* = 1), *E. coli* (*n* = 29), *K. pneumoniae* (*n* = 17), *K. quasipneumoniae* (*n* = 3).

### Carbapenem Resistance Mechanisms

The WGS data confirmed that none of the isolates harbored any known carbapenemase genes. However, we found novel deletions, including the deletion of 24 amino acids from Met1 to Val24 (M1_V24del) in the *ompC* gene of all the *E. coli* isolates (29/29) (Table 3). Previously known substitutions in the *ompC* gene mediating carbapenem resistance were also detected among *E. coli* isolates (D192G/K in 17/29 isolates; N47D in 5/29 isolates) but, no mutations associated with carbapenem resistance were detected in the *ompF* gene. Among the *Klebsiella* isolates, we detected a HYTH insertion between amino acid Met233 and Thr234 (M233_T234insHYTH) (10/20) insertion in the *ompK37*, substitution A217S (15/20) and N218H (6/20) in *ompK36*, and substitution I70M (20/20), I128M (20/20), N230G (10/20) and T261A (1/20) in *ompK37* (Table 4).

**TABLE 3:** Characteristics and resistance mechanisms associated with concurrent carbapenem-colistin among *Enterobacteriaceae* isolated from animals in Nigeria.

Sample source	ID no	Species	*mcr*- genes	Mutations and genetic alterations mediating								
				mgrB	*ccrB*	*etpA*	*arnT*	*ompC*	*marR*	*ramR*	*ompK36*	*ompK37*
Cattle Rectal swab	B14	*E. coli*	–	M1V	–	–	A278T	M1_V24del, G29S, D39N, G40A, L41K, D46S, Q54T, Y74F	M1V	–	–	–
	B22	*E. coli*	–	M1V	–	–	–	M1_V24del	M1V	–	–	–
Camel rectal swab	C40	*E. coli*	–	M1V	–	V336M		M1_V24del, D192G	M1V	–	–	–
Pig rectal swab	E41	*E. coli*	–	M1V	–	–	–	M1_V24del, D192G	M1V	–	–	–
	L6	*E. coli*	–	M1V	–	–	–	M1_V24del, D192G	M1V	–	–	–
	L13	*E. coli*	–	M1V	–	–	–	M1_V24del, D192G	M1V	–	–	–
	L15	*E. coli*	–	M1V	–	–	–	M1_V24del, D192G	M1V	–	–	–
	L16	*E. coli*	–	M1V	–	–	–	M1_V24del, D192G, N228_T229insGSYTSNGV	M1V	–	–	–
	L17	*E. coli*	–	M1V	–	–	–	M1_V24del, D192G	M1V	–	–	–
	L20	*E. coli*	–	M1V	–	T411A	T157A	M1_V24del	M1V	–	–	–
Poultry cloacal swab	L3	*E. coli*	–	M1V	–	–	–	M1_V24del	M1V	–	–	–
	L18	*K. pneumonia*	–	M1V	M1_L56del, W140S, N141H, N195S	–	–	–	–	M1V	A217S, N218H	I70M, I128M
	L22	*E. coli*		M1V	–	–	–	M1_V24del, D192G	M1V	–	–	–
	L23	*E. coli*	*mcr*-*1*	M1V	–	–	–	M1_V24del, N47D, D192K	M1V	–	–	–
	L25	*E. coli*		M1V	–	–	–	M1_V24del, N47D, D192K	M1V	–	–	–
	L26	*E. coli*	*mcr*-*1*	M1V	–	–	L485F	M1_V24del	M1V	–	–	–
	L27	*E. coli*	*mcr*-*1*	M1V	–	–	–	M1_V24del, D192G, N228_T229insGSYTSNGV	M1V	–	–	–
	L28	*E. coli*		M1V	–	–	–	M1_V24del, D192G	M1V	–	–	–
	L29	*E. coli*	*mcr*-*1, mcr*-*5*	M1V	–	–	–	M1_V24del, N47D, D192K	M1V	–	–	–
	L31	*E. coli*		M1V	–	–	–	M1_V24del	M1V	–	–	–
Liver from sick birds	L36	*E. coli*	*mcr*-*1*	M1V	–	–	–	M1_V24del	M1V	–	–	–
	L38	*Citrobacter werkmanii*	*mcr*-*1*	M1V	–	–	–	–	M1V	–	–	–
	L39	*E. coli*	*mcr*-*1*	M1V	–	–	–	M1_V24del, N47D	M1V	–	–	–
	L40	*E. coli*	*mcr*-*1*	M1V	–	–	–	M1_V24del, A230_A231insYYISNGVAR	M1V	–	–	–
	L41	*E. coli*	–	M1V	–	–	W100M	M1_V24del, K173T, N176_T186del, G190E, D192G, D225W	M1V	–	–	–
	L43	*E. coli*	–	M1V	–	–	–	M1_V24del, N47D, D192G	M1V	–	–	–

*M1V: Depicts a substitution of the amino acid methionine (M) by valine (V) at position 1; M1_V24del: depicts a loss/deletion of the first 24 amino acids, Methionine (M) at position 1 through Valine (V) at position 24; N228_T229insGSYTSNGV: depicts an insertion of 8 amino acids GSYTSNGV between position 228 and 229.*

**TABLE 4:** Characteristics and resistance mechanisms associated with concurrent carbapenem-colistin among *Enterobacteriaceae* isolated from human sources in Nigeria.

Sample source	ID no	Species	*mcr* gene	Mutations and genetic alterations mediating							
				*mgrB*	*ccrB*	*arnT*	*ompK36*	*ompK37*	*ompC*	*ramR*	*marR*
Human clinical sample (stool)	H2	*K. pneumonia*	*mcr*-*1, mcr*-*8*	M1V	N195S	–	A217S, N218H	I70M, I128M	–	M1V	–
	H6	*K. pneumonia*	*mcr*-*8*	M1V	N195S	–	A217S	I70M, I128M, N230G, M_233T234insHYTH	–	M1V	–
	H34	*E. coli*	–	M1V	–	–	–	–	M1_V24del	–	M1V
	H35	*E. coli*	–	M1V	–	–	–	–	M1_V24del, D192G	–	M1V
	H36	*K. pneumonia*	–	M1V	M1_A52del, W140L, N141I	–	A217S	I70M, I128M	–	M1V	–
	H41	*E. coli*	–	M1V	–	–	–	–	M1_V24del, R267L	–	M1V
	H50	*K. quasipneumoniae*	–	M1V	M1_A59del, W140S, N141H, N195S	–	A217S, N218H	I70M, I128M	–	M1V	–
Human clinical sample (urine)	H4	*K. pneumonia*	–	M1V	N195S	–	–	I70M, I128M, N230G, M233_T234insHYTH	–	M1V	–
	H5	*K. pneumonia*	–	M1V	N195S	–	–	I70M, I128M, N230G, M233_T234insHYTH	–	M1V	–
	H7	*K. pneumonia*	*mcr*-*8*	M1V	N195S	G164S	–	I70M, I128M, N230G, M233_T234insHYTH	–	M1V	–
	H23	*K. pneumonia*	–	M1V	N195S	–	–	I70M, I128M, N230G, M233_T234insHYTH	–	M1V	–
	H25	*K. pneumonia*	–	M1V	N195S	–	–	I70M, I128M, N230G, M233_T234insHYTH	–	M1V	–
	H26	*K. pneumonia*	–	M1V	N195S	–	–	I70M, I128M, N230G, M233_T234insHYTH	–	M1V	–
	H29	*E. coli*	–	M1V	–	–	–	–	M1_V24del	–	M1V
	H30	*K. quasipneumoniae*	–	M1V	M1_A59del, W140S, N141H, N195S	–	A217S, N218H	I70M, I128M	–	M1V	–
	H31	*K. pneumonia*	*mcr*-*1, mcr*-*8*	M1V	N195S	–	A217S, N218H	I70M, I128M	–	M1V	–
	H39	*K. pneumonia*	*mcr*-*8*	M1V	N195S	–	–	I70M, I128M, T261A	–	M1V	–
	H40	*K. pneumonia*		M1V	M1_A59del, W140L, N141H	–	A217S, N218H	I70M, I128M	–	M1V	–
	H45	*K. pneumonia*	*mcr*-*1*	M1V	N195S	–	A217S	I70M, I128M, N230G, M233_T234insHYTH	–	M1V	–
	H46	*K. pneumonia*	–	M1V	N195S	–	A217S	I70M, I128M, N230G, M233_T234insHYTH	–	M1V	–
	H47	*K. pneumonia*	–	M1V	N195S	–	A217S	I70M, I128M, N230G, M233_T234insHYTH	–	M1V	–
	H48	*K. pneumonia*	–	M1V	N195S	–	A217S	I70M, I128M, N230G, M233_T234insHYTH	–	M1V	–
	H49	*E. coli*	–	M1V		–	–	I70M, I128M, N230G, M233_T234insHYTH	M1_V24del, D192G, A231_Y232insNGYGER	–	M1V
Environon-mental sample (sink)	H22	*K. quasipneumoniae*	–	M1V	N195S	–	A217S	I70M, I128M, N230G, M233_T234insHYTH	–	–	–

*M1V: Depicts a substitution of the amino acid methionine (M) by valine (V) at position 1; M1_A52del: depicts a loss/deletion of the first 52 amino acids i.e., Methionine (M) at position 1 through Alanine (A) at position 52; M233_T234insHYTH: depicts an insertion of 4 amino acids HYTH between position 233 and 234.*

Multiple substitutions were detected in the global regulator proteins *marR* and *ramR*, but not in the regulators *AcrR, MarA, RamA*, and *SoxR*. Only the M1V substitution in *E. coli marR* and *K. pneumoniae ramR* was associated with carbapenem resistance (Tables 3, 4).

### Colistin Resistance Mechanisms

Sixteen (one *C. werkmanii*, eight *E. coli*, and seven *K. pneumoniae* isolates) of the 50 carbapenem-colistin resistant isolates harbored the plasmid mediated colistin resistant genes: *mcr*-1 (*n* = 9), *mcr*-1 and +*mcr*-5 (*n* = 1), *mcr*-8 (*n* = 4), *mcr*-1, and +*mcr*-8 (*n* = 2) (Tables 3, 4). No point mutations associated with colistin resistance were detected in the *pmrABC* and *phoPQ* genes. Alterations in the *mgrB* were detected in all the isolates viz *E. coli* (29/29), *Klebsiella* (20/20), and *Citrobacter* (1/1) (Tables 3, 4). Novel substitutions were found in some the *E. coli* isolates, including V336M and T411I in the *etpA* and A278T, T157A, L485F, and W100M in the *arnT* (Table 3). The majority of the *Klebsiella* isolates harbored the N195S amino acid substitution (18/20), and some harbored the N141I/H (5/20) and W140L/S (5/20) substitutions in the *ccrB*, which were previously reported to mediate colistin resistance (Cheng et al., 2016). Finally, novel mutations as predicted to mediate colistin resistance, G164S in *arnT*, and the M1_L56del deletion were detected in one *Klebsiella* isolate (Tables 3, 4).

### Resistome Other Than Colistin and Carbapenem Resistance of the Isolates

The 50 sequenced isolates harbored a plethora of resistant genes including β-lactamases (*bla*_*CTX–M*_, *bla*_*SHV*_, bla_*OXA–*__1_, *bla*_*TEM–*__1_, *bla*_*CMY*_, *bla*_*DHA*_), aminoglycosides [*aac(3)*-*IIa*, *aac(6′)*-*Ib3, aac(3)*-*VIa, aph(3′)*-*Ia*, *aph(3″)*-*Ib*, *aph(6)*-*Ic, aph(6)*-*Id*, *armA*, *aadA1, aadA2b, aadA5, aac(6′)*-*Ib*-*cr*], fluoroquinolones [*qepA1, qepA4, oqxAB, qnrS1, qnrS13, qnrB4, qnrB17, qnrB19, qnrB34*, *aac(6′)*-*Ib*-*cr*], sulfonamides (*sul1*, *sul2, sul3*), trimethoprim (*dfrA1, dfrA10, dfrA12, dfrA14, dfrA15, dfrA17 dfrB4*), phenicols (*catA2, catB3, cmlA1, floR*), tetracyclines [*tet(A), tet(B), tet(D), tet(J), tet(M)*] as well as mutations in *acrR, gyrA*, and *parC* for fluoroquinolones and *ramR* for tetracyclines particularly tigecycline (Tables 1, 2).

Genes *bla*_*TEM*_ (15/30) and *bla*_*CTX*__–_*_*M*_* (14/30) were found to be the predominant beta-lactamases among the *E. coli* isolates (Table 1) while *bla*_*SHV*_ was frequently observed among the *Klebsiella* isolates (15/20) (Table 2). Some isolates (19/50) harbored more than one *bla* in different combinations. The single *Citrobacter* isolate in the study harbored the only AmpC type *bla* detected i.e., CMY-98 genotype (Table 1). Fluoroquinolone resistance was mostly mediated by plasmid mediated quinolone resistance *qnr* (16/29) and chromosomal mutations (S83L, D87N) of the *gyrA* (15/29) in the *E. coli.* Amongst the *Klebsiella* isolates, quinolone resistance was mostly due to a mutation of the *acrR* gene (18/20). Seven isolates harbored the *aac(6)*-*Ib*-*cr* gene which confers resistance to both aminoglycosides and fluoroquinolones.

Gene *tet(A)* (20/29) was the most common tetracycline resistance gene carried by the *E. coli* isolates while *tet*(A) (5/20) and *tet(D)* (*n* = 5/20) were the most common among the *Klebsiella* species. The *tet(M)* commonly restricted to Gram-positive bacteria was detected in one *E. coli* and a *K. pneumoniae* isolated from a cloacal swab from poultry. Additionally, one of the *Klebsiella* isolates (L18) harbored the *ramR* mutation (A19V) responsible for tigecycline resistance.

### Genetic Diversity

Multilocus sequence and phylogroup typing showed that the isolates were polyclonal and genetically diverse (Supplementary Tables 2, 3). The *E. coli* isolates clustered within six of the eight known phylogroups: A, B1, B2, C, D, E with phylogroup B1 been the predominantly occurring (12/29) based on the Clermont typing profile. MLST assigned the isolates into 23 sequence types (STs) (and one1 unknown ST) with four of the STs occurring more than once: ST191 (*n* = 3), ST224 (*n* = 2), ST410 (*n* = 3), and ST2485 (*n* = 3) (Supplementary Table 2). cgMLST assigned them into 24 different cgSTs due to genetic differences between the two ST224 strains which were identified as cgST72904 (isolate L17) and cgST38401 (isolate L22) (Supplementary Table 3).

*In silico* serotyping using the WGS data assigned the isolates into 23 serotypes, O128ab/ac:H20 (*n* = 2). Although the “O”-group antigen of eight isolates were unknown, the isolates belonged to the H9 (*n* = 3), H21 (*n* = 1), H23 (*n* = 1), H30 (*n* = 1), H37 (*n* = 1), and H45 (*n* = 1).

Similarly, the *Klebsiella* isolates were distributed into 14 different multilocus sequence types (STs) with three STs occurring more than once: ST45 (*n* = 2), ST307 (*n* = 3), and ST340 (*n* = 3). We were however unable to infer the ST for one of the *K. quasipneumoniae* isolate (Supplementary Table 3). *In silico* serotyping of the *Klebsiell*a isolates assigned them into 15 different serotypes with the most commonly occurring serotype being KL102:O2v2 (*n* = 4).

### Plasmidome and Virolome

Thirty different plasmid replicon types were detected via analyses of the WGS data using PlasmidFinder (Supplementary Tables 2, 3). The majority of the isolates harbored at least two plasmid replicon types with the most abundant being the IncFIB (AP001918) in *E. coli* (*n* = 17) and IncFIB(K) in *Klebsiella* (*n* = 16). Other dominant replicon types were IncFII and IncFIA in *E. coli* (*n* = 9 each), IncFII(K) and IncR in *Klebsiella* (*n* = 11 each). No plasmids were detected in three *E. coli* and two *Klebsiella* isolates.

Although, the *mcr*-1 was located in a unique contig that had the same sequence as the IncX4 plasmid backbone, no other resistance genes were found on the IncX4 plasmid in this study. Other resistance genes harbored on plasmids include *sul2* and *aph(3″)*-*Ib* on *IncQ1*, *blaTEM*-*1B* on *IncX1*, *blaTEM*-*1C* on *IncFII(K).*

A total of 42 different virulence genes were detected across the 50 isolates (Supplementary Tables 2, 3). Carriage of extra-intestinal pathogenic *E. coli* (ExPEC) virulence associated genes (VAGs): *cva, cvi*, *hylF*, *iroN*, *iss*, *iutA*, *ompT*, *sitA*, *traT* was widespread among the *E. coli* isolates (Supplementary Table 2). Pathotype specific VAGs including *astA* (EAEC), *afaD, hlyA, ihA, fyuA* (UPEC), *vat* (APEC), and *papC* (APEC/UPEC) were also detected (Supplementary Table 2).

### Conjugation Assay

The conjugation assay confirmed transfer of the colistin resistant determinant from two isolate (H2 and L40) to the recipient *E. coli* J53 with conjugation frequencies of 3.4 × 10^–1^ cfu/recipient cfu and 4.6 × 10^–1^ cfu/recipient cfu, respectively. None of the isolates transferred the carbapenem resistant determinant. The colistin MIC for the two transconjugants was 4 μg/mL compared with 0.5 μg/mL for the *E. coli* J53. Furthermore, the transconjugants expressed two resistant phenotypes (L40: AMC-AMX-CIP-CEF-DOX-GEN; H2: AMC-AMX-CEF-GEN).

## Discussion

The last two decades have witnessed a significant rise in infections caused by multidrug resistant *Enterobacteriaceae* and resulted in an increase in the use of carbapenems and colistin as last resort drugs (Peyclit et al., 2019). Concurrent resistance to last resort drugs represents a serious health concern globally (Chaudhary, 2016; Serwecinska, 2020) and our study has provided evidence of high levels of concurrent resistance to colistin and carbapenem in *Enterobacteriaceae* in Nigeria with a prevalence of 9.1%. This is of major concern as Nigeria is a low-income country with minimal antimicrobial surveillance. This also indicates the emergence and establishment of potentially pandrug resistant strains, and creates limitations in the ability to control common infections.

While transferable mobile genetic elements such as *mcr*- and carbapenemase genes have generated much interest and are the target of most studies, our results show that concurrent carbapenem–colistin resistance in the *E. coli* and *K. pneumoniae* isolates is also linked to previously reported and novel mechanisms. These mechanisms include chromosomal mutations/disruptions affecting regulatory and non-regulatory genes controlling efflux-reflux pumps and membrane permeability. Such mutations/disruptions may play a greater role in resistance to carbapenem and colistin resistance than previously suspected.

Surprisingly, we found no evidence for carbapenemase producing genes in both our phenotypic and genomic investigations (WGS). This is in contrast to some studies on resistance in the region that reported carbapenemase genes such as *bla*_*NDM*_ and *bla*_*OXA*__–_*_181_* are widespread (Jesumirhewe et al., 2017; Olalekan et al., 2020; Olowo-Okere et al., 2020; Shettima et al., 2020).

Consistent with previous reports on their role in colistin/carbapenem resistance, we detected the plasmid mediated colistin resistance genes; *mcr*-1 *mcr*-5, and *mcr*-8, and alterations/mutations in the *mgrB, ccrB*, that mediates colistin resistance as well as alterations/mutations in the *OmpC, OmpK36, OmpK37* which mediates carbapenem resistance (Olaitan et al., 2014; Poirel et al., 2015; Cheng et al., 2016). We also identified multiple potential novel mutations/alteration associated with colistin resistance: *arnT* (W100M, T157A, G164S, A278T, L485F), *etpA* (V336M, T411H), *pmrB* (G164S, R256G), *ccrB* (M1_L56del, M1_KA52del, M1_A78del, M1_M59del) and carbapenem resistance: *ompC* (M1_V24del, K173T, N228_T229insGSYTSNGV, A231_Y232insNGYGER, A230_A231insYYISNGVAR), ompK37 (K27Q, D28Q, G29V, N30G, K31S, D33T, M1_Y25del, M233_T234insHYTH). Colistin resistance is mediated by LPS modification, which is encoded by the *pmrHFIJKLM* operon and the *pmrC* locus, and regulated by PhoPQ and PmrAB. This modification decreases the negative charge of the outer membrane, reducing its interaction with colistin (Trimble et al., 2016). Alterations in the genes of the two component regulatory systems (2CRS) particularly *mgrB, phoP/phoQ, pmrA, pmrB, pmrC*, and *crrABC* mediate colistin resistance (Wright et al., 2015; Cheng et al., 2016). Genetic alteration in the *mgrB* gene, which was common among the isolates, results in a disruption of the negative feedback loop of the PhoP/PhoQ, overexpression of the PhoP-regulated genes leading to the up-regulation of the *pmrHFIJKLM* operon and an abnormally high levels of lipid A modification, and ultimately a low susceptibility to colistin. Similarly, *crrB* mutations consistent with those detected in this study were shown to mediate reduced colistin susceptibility (Cheng et al., 2016) via induction of CrrC expression, which induces an elevated expression of the *pmrHFIJKLM* operon and *pmrC* via the PmrAB two-component system, loss of regulation of the *crrAB* gene that encodes a glycosyltransferase-like protein, which in turn leads to modification of lipid A and increased autophosphorylation of *ccrB* which leads to colistin resistance (Wright et al., 2015; Cheng et al., 2016; Aghapour et al., 2019). Porins are outer membrane proteins associated with the modulation of cellular permeability and antibiotic resistance. OmpF and *OmpA* in *E. coli* and OmpK35/36/37 in *Klebsiella* play a major role in antibiotic transport into the bacteria cell. Alterations in the gene encoding these proteins have been reported to infer with protein configuration and thus entry of antibiotics, particularly carbapenems (Fernández and Hancock, 2012). We hypothesized that the observed disruptions in these gene as observed among our isolates and identified as deleterious by PROVEAN may be responsible for the high carbapenem MIC and resistance in this study.

While the significance of these mutations and their impact on the MIC and resistance to carbapenem and colistin will require expression level analyses including transcriptomics and complementation assays, we hypothesize that the observed concurrent carbapenem–colistin resistance resulted from a combination of the chromosomal mutations/alterations in the *mgrB* (M1V) *ompC* (M1_V24del)*ompK37* (I70M, I128M) and the regulatory efflux pump genes (*marR* (M1V), and *ramR* (M1V). Other studies have reported that mutations in the efflux system, particularly the global regulator *marR* and *ramR* as found in our study, β-lactamase production, and porin deficiency could play major roles in carbapenem resistance (Girlich et al., 2009; Findlay et al., 2012; Shin et al., 2012; Adler et al., 2013; Tsai et al., 2013; Chetri et al., 2019, 2020). An important finding in this study that we also wish to highlight, is the high probability for an isolate that is colistin resistant to also be carbapenem resistant, and vice versa.

Reports emanating particularly from Asia demonstrate a significant reduction in the rate of colistin resistant isolates due to the effect of the ban on colistin use in the wake of the detection of the plasmid mediated *mcr*-*1* gene (Walsh and Wu, 2016; EMA/AMEG, 2021; Usui et al., 2021; Wang Y. et al., 2020). Similarly, countries such as the United States, Canada, and United Kingdom which have never approved colistin usage in animal production have continually reported lower rates of colistin resistant strains (EMA/AMEG, 2021). However, this is not the situation in Nigeria and other African countries where there is a rise in the numbers of colistin resistant strains, as demonstrated in this study. No such ban or regulation on colistin use exist in Nigeria where its use is currently widespread and is a common active ingredient in most antibiotic combinations/preparation used in livestock production for the purpose of prophylaxis and therapy. Although carbapenem is rarely used in food animals in Nigeria, the inappropriate use of colistin has been shown to provide selective pressure for the emergence of colistin and multidrug (including carbapenem) resistant strains (Napier et al., 2013). We, therefore, hypothesize that the high rates of co-resistance observed in our study may be connected to the widespread use of colistin, particularly in livestock production in Nigeria.

Majority of the isolates expressed multidrug resistance profiles, including high resistance to amoxicillin-clavulanic acid (98%), fluoroquinolones (78%), and gentamicin (48%) which are widely used in the treatment of infections in Nigerian hospitals. The high resistance rates we recorded are in accordance with data in other reports on human and animal isolates from Nigeria and Sub-Saharan Africa (Ojo et al., 2016; Chah et al., 2018; Aworh et al., 2019; Olalekan et al., 2020; Shettima et al., 2020). Most of our isolates also carried the *bla*_*CTX–M–*__15_ which is consistent with the increasing reports of this genotype from animal and human sources in Nigeria (Chah et al., 2018; Okpara et al., 2018; Olowo-Okere et al., 2020). There is thus growing evidence that this genotype is expanding rapidly and might become the dominant mechanism mediating resistance to the β-lactams.

The significant clonal diversity i.e., polyclonality observed amongst our isolates is consistent with previous reports, particularly those of carbapenem and colistin resistant isolates in Nigeria where the population structure is diverse (Ngbede et al., 2020; Olalekan et al., 2020). Some of the animal and human isolates in our study had similar STs with previously reported ″high-risk″ clones including *K. pneumoniae* ST11, ST17, ST45, ST340 (human isolates) and *E. coli* ST58, ST744, ST410 (animal isolates). These high risk STs are known for their global dissemination, ease of transmission between different hosts, ability to cause disease and acquire genetic determinants such as virulence factors, epidemic plasmids and antibiotic resistance that provide them with a competitive advantage over other bacterial clones (Lee et al., 2016; Roer et al., 2018; Feng et al., 2019; Nadimpalli et al., 2019; Patiño-Navarrete et al., 2020). The *E. coli* ST58, ST744, ST410 virulence profile categorized them to the UPEC, EAEC and DAEC pathotypes. Similarly, the ST11 and ST340 detected in our human isolates are closely related to ST258, all belonging to clonal complex CC258 which has been associated with outbreaks, pandemics and mass dissemination of KPC *K. pneumoniae* (Netikul and Kiratisin, 2015; Sui et al., 2018; Cienfuegos-Gallet et al., 2019; Fu et al., 2019; Zhao et al., 2019).

## Conclusion

In this study, we report evidence for the occurrence of multidrug resistant *Enterobacteriaceae* with concurrent carbapenem-colistin resistance in 9.1% of the isolates. The genetic mechanism underlying this concurrent resistance phenotype was majorly novel and previously known chromosomal alterations (deletion, insertions, and substitutions). The plasmid-mediated colistin resistance gene *mcr*- in combination with these chromosomal alterations accounted for colistin resistance in few of the isolates. Some of the *E. coli* and *Klebsiella* isolates expressing concurrent carbapenem-colistin resistance in this study belonged to the internationally recognized “high-risk” clones. The combination of diverse drug resistance genes and sequence types highlight the considerable genome plasticity and polyclonality that characterize the population structure of both clinical and non-clinical colistin and CRE in Nigeria. The polyclonality might create considerable problems during outbreak tracing and source attribution.

## Data Availability Statement

The raw data supporting the conclusions of this article will be made available by the authors, without undue reservation.

## Ethics Statement

The studies involving human participants were reviewed and approved by Research and Ethics Committee of the facilities (FMH/FMC/MED.108/VOL.I/X and BSUTH/MKD/HREC/2013B/2018/0027). Written informed consent for participation was not required for this study in accordance with the national legislation and the institutional requirements.

## Author Contributions

EN and CW: conceptualization and funding acquisition. EN, FA, AP, and CW: methodology. EN, AMA, SD, AAA, CA, PA, LM, NM, and MA: sampling/investigation. EN, FA, AP, AK, YY, and PK: data curation. EN, MA, and CW: supervision. EN and FA: writing—original draft preparation. EN, YY, PK, OL, PB, and CW writing—review and editing. All authors read and agreed to the published version of the manuscript.

## Conflict of Interest

The authors declare that the research was conducted in the absence of any commercial or financial relationships that could be construed as a potential conflict of interest.

## Publisher’s Note

All claims expressed in this article are solely those of the authors and do not necessarily represent those of their affiliated organizations, or those of the publisher, the editors and the reviewers. Any product that may be evaluated in this article, or claim that may be made by its manufacturer, is not guaranteed or endorsed by the publisher.

## Footnotes

1
http://cab.cc.spbu.ru/quast/

2
https://cge.cbs.dtu.dk/services/KmerFinder/

3
https://pubmlst.org/bigsdb

4
https://cge.cbs.dtu.dk/services/cgMLST

5
http://clermontyping.iame-research.center/index.php

6
https://cge.cbs.dtu.dk/services/SerotypeFinder/

7
http://kaptive.holtlab.net/

8
https://cge.cbs.dtu.dk/services/ResFinder/

9
https://card.mcmaster.ca/analyze/rgi

10
https://cge.cbs.dtu.dk/services/PlasmidFinder/

11
https://cge.cbs.dtu.dk/services/MobileElementFinder/

12
http://provean.jcvi.org/seq_submit.php
