# Supplementary material for: Concurrent Resistance to Carbapenem and Colistin Among Enterobacteriaceae Recovered From Human and Animal Sources in Nigeria Is Associated With Multiple Genetic Mechanisms
Source: Front Microbiol. 2021 Oct 6;12:740348. doi: 10.3389/fmicb.2021.740348 (PMC8528161; doi:10.3389/fmicb.2021.740348)
Supplement: Supplementary file 1 [file Data_Sheet_1.PDF]

Supplementary Table S1: Colistin and carbapenem minimum inhibitory concentration (MIC) for *Enterobacteriaceae* expressing concurrent resistance to carbapenem and colistin, Nigeria

Sample ID	Species	Host	Sample type	Minimum Inhibitory concentration (MIC) µg/ml			
				**Colistin**	**Meropenem**	**Ertapenem**	**Imipenem**
H2	*K. pneumoniae*	Human	Stool	>64	32	2	2
H4	*K. pneumoniae*	Human	Urine	>64	32	2	8
H5	*K. pneumoniae*	Human	Urine	>64	32	16	12
H6	*K. pneumoniae*	Human	Stool	>64	32	2	4
H7	*K. pneumoniae*	Human	Urine	>64	32	16	12
H22	*K. quasipneumoniae*	HE	Sink	32	32	16	12
H23	*K. pneumoniae*	Human	Urine	32	8	16	12
H25	*K. pneumoniae*	Human	Urine	32	32	16	12
H26	*K. pneumoniae*	Human	Urine	>64	6	16	8
H29	*E. coli*	Human	Urine	16	4	2	2
H30	*K. quasipneumoniae*	Human	Urine	16	32	16	12
H31	*K. pneumoniae*	Human	Urine	>64	8	8	12
H34	*E. coli*	Human	Stool	16	16	8	12
H35	*E. coli*	Human	Stool	> 64	2	2	2
H36	*K. pneumoniae*	Human	Stool	16	16	2	8
H39	*K. pneumoniae*	Human	Urine	>64	16	16	2
H40	*K. pneumoniae*	Human	Urine	>64	32	16	12
H41	*E. coli*	Human	Stool	>64	8	16	8
H45	*K. pneumoniae*	Human	Urine	>64	32	16	12
H46	*K. pneumoniae*	Human	Urine	>64	32	16	8
H47	*K. pneumoniae*	Human	Urine	>64	16	16	12
H48	*K. pneumoniae*	Human	Urine	>64	16	16	12
H49	*E. coli*	Human	Urine	>64	32	2	12
H50	*K. quasipneumoniae*	Human	Stool	16	16	16	12
B14	*E. coli*	Cattle	RS	16	32	16	12
B22	*E. coli*	Cattle	RS	16	32	16	12
C40	*E. coli*	Camel	RS	>64	32	8	8
E41	*E. coli*	Pig	RS	>64	8	16	8
L3	*E. coli*	Poultry	CS	>64	32	2	8
L6	*E. coli*	Pig	RS	>64	4	2	2
L13	*E. coli*	Pig	RS	32	32	16	4
L15	*E. coli*	Pig	RS	>64	8	16	12
L16	*E. coli*	Pig	RS	16	32	8	4
L17	*E. coli*	Pig	RS	16	16	16	12
L18	*K. pneumoniae*	Poultry	CS	16	8	16	8
L20	*E. coli*	Pig	RS	16	32	16	8
L22	*E. coli*	Poultry	CS	16	32	8	8
L23	*E. coli*	Poultry	CS	16	32	16	12
L25	*E. coli*	Poultry	CS	16	16	16	12
L26	*E. coli*	Poultry	CS	16	32	16	4
L27	*E. coli*	Poultry	CS	16	32	16	4
L28	*E. coli*	Poultry	CS	16	32	2	4
L29	*E. coli*	Poultry	CS	16	8	16	12
L31	*E. coli*	Poultry	CS	16	32	2	4
L36	*E. coli*	Poultry	VL	16	32	4	8
L38	*E. coli*	Poultry	VL	16	32	16	12
L39	*E. coli*	Poultry	VL	16	4	2	4
L40	*E. coli*	Poultry	VL	16	16	16	4
L41	*E. coli*	Poultry	VL	16	8	8	8
L43	*E. coli*	Poultry	VL	64	32	4	12

RS: Rectal swab, CS: Cloacal swab, VL: Vicera-Liver

Supplementary Table S2: Genetic diversity and virulence profile of *E. coli* isolated from animals and humans in Nigeria

ID	MLST	cgMLST	Phylo-group	Serotype	Virulence profile	Plasmid Profile	Pathotype
B14	ST48	114697	B1	O:H45	*hra, iss, ipfA, terC*	IncQ1, IncFIA, IncFIB(AP001918), IncFIB(K)	ExPEC
B22	ST215	87497	A	O68:H12	*capU, gad, terC*		ExPEC
C40	ST46	121659	A	O8:H4	*iss, sitA, terC*		ExPEC
E41	ST4977	3627	A	O188:H26	*gad, iss, ipfA, terC*	IncFIB(K)	ExPEC
H29	ST210	11840	B1	O96:H19	*fyuA, ihA, irp2, iutA, ipfA, mchB, mchC, mchF, terC*		UPEC
H34	ST156	100565	B1	O174:H28	*gad, hra, iss, ipfA, terC*	IncFIA(HI1), Col440I, IncFIB(K)	ExPEC
H35	ST2006	135368	B1	O9a:H23	*astA, iss, ipfA, terC, traT*	Col156, IncFIB(AP001918), IncFII(pAMA1167-NDM-5)	EAEC
H41	ST182	102234	E	O:H30	*chuA, eilA, terC*	IncY	UPEC
H49	**ST58**	89021	B1	O8:H25	*cia, cvaC, etsC, fyuA, hlyA, iroN, irp2, iss, iucC, iutA, ipfA, mchF, ompT, sitA, terC, traT*	IncQ1, IncFIB(AP001918), IncFII	UPEC
L3	ST196	40677	B1	O115:H7	*iss, ipfA, sitA, terC*	IncY	ExPEC
L6	**ST410**	121598	C	O:H9	*afaA, afaB, afaC, afaD, afaE, fyuA, gad, hra, irp2, iucC, iutA, papA_F13, papC, senB, sitA, terC*	Col156, IncFIA, IncFIB(AP001918), IncFII	DEAC/ ExPEC
L13	**ST410**	121598	C	O:H9	*afaA, afaB, afaC, afaD, afaE, fyuA, gad, hra, irp2, iucC, iutA, papA_F13, papC, senB, sitA, terC*	Col156, IncFIA, IncFIB(AP001918), IncFII	DAEC/ ExPEC
L15	**ST410**	121598	C	O:H9	*afaA, afaB, afaC, afaD, afaE, fyuA, gad, hra, irp2, iucC, iutA, papA_F13, papC, senB, sitA, terC*	Col156, IncFIA, IncFIB(AP001918), IncFII	DAEC/ ExPEC
L16	ST226	44836	A	O141ab/ac:H5	*gad, terC*	IncY	ExPEC
L17	ST244	72904	B1	O8:H23	*ipfA, terC*		ExPEC
L20	ST2179	133214	B1	O9a:H9	*cib, cma, cvaC, etsC, fyuA, gad, hlyF, hra, iroN, irp2, iss, iucC, iutA, ipfA, ompT, sitA, terC, traT*	IncFIB(AP001918), IncFIC(FII)	UPEC/ ExPEC
L22	ST224	38401	B1	O:H23	*afaD, cma, cvaC, gad, hlyF, iha, ipfA, nleA, ompT, terC, sitA*	IncFIB(AP001918), IncX1, IncI1, IncI2	UPEC/ ExPEC

L23	ST2485	131215	D	O15:H45	*astA, chuA, cib, cma, cvaC, eilA, hlyF, iroN, iss, iuC, iutA, ompT, sitA, terC, traT*	IncFIB(AP001918), IncFIC(FII), IncX4, IncFIA, IncX1	EAEC/ ExPEC
L25	ST2485	131215	D	O15:H45	*astA, chuA, cib, cma, cvac, eilA, hlyF, iroN, iss, iucC, iutA, ompT, sitA, terC, traT*	IncFIB(AP001918), IncFIC(FII), IncX4, IncFIA, IncX1	EAEC/ ExPEC
L26	ST115	32494	B1	O155:H7	*cib, cma, cvaC, fyuA, hlyF, hra, iroN, irp2, iss, ipfA, ompT, terC*	IncFIB(AP001918), IncFII, IncI1-I(Gamma), IncX4, IncY	ExPEC
L27	**ST744**	119414	A	O101:H9	*astA, cma, cvaC, gad, hlyF, iroN, iss, ompT, sitA, terC, traT*	IncFIB(AP001918), IncFII, IncX4, p0111, IncX1	EAEC/ ExPEC
L28	ST1673	133799	B1	O:H21	*cib, cma, cvaC, etsC, hlyF, iroN, iss, iucC, iutA, ompT, sitA, terC, traT, tsh*	IncQ1, IncFIB(AP001918), IncI1, IncFIC(FII)	APEC/ ExPEC
L29	ST2485	131215	D	O15:H45	*air, astA, chuA, cib, cma, cvaC, eilA, hlyF, iroN, iss, iucC, iutA, ompT, sitA, terC, traT*	IncFIB(AP001918), IncFIC(FII), IncX4, IncFIA, IncX1	EAEC/ ExPEC
L31	ST6061	61379	A	O21:H52	*cea, celb, cib, cma, cvaC, gad, hlyF, hra, iha, iroN, iss, kpsE, kpsM111_K96, ompT, papA_F20, papC, sitA, terC, traT*	IncFIB(AP001918), IncFIC(FII), Col156, IncN, p0111	APEC/ ExPEC
L36	ST191	133726	A	O128ab/ac:H20	*cma, cvaC, gad, hlyF, iroN, iss, ipfA, ompT, sitA, terC, traT*	IncFIB(AP001918), IncFII, Col3M, p0111, IncX1	APEC/ ExPEC
L39	ST2954	66635	E	O160:H9	*chuA, eilA, gad, hlyF, iss, ompT, sitA, terC, traT*	IncX4, IncX1, IncFIB(AP001918), IncFII, IncFIB(pLF82), IncQ1	APEC/ ExPEC
L40	ST191	133726	A	O128ab/ac:H20	*cma, cvaC, hlyF, iroN, iss, ipfA, ompT, sitA, terC, traT*	IncFIB(AP001918), IncFII, IncX4, p0111, IncX1	APEC/ ExPEC
L41	Unknown	39798	A	O:H37	*gad, terC*	p0111, IncFIB(K), IncHI1B(CIT)	ExPEC
L43	ST929	25427	B2	O156:H14	*chuA, cia, etsC, fyuA, hlyF, ibeA, iroN, irp2, iss, kpsE, kpsM11, ompT, papC, pic, sitA, terC, traT, usp, vat, yfcV*	IncX1, IncFII, IncFII(pHN7A8), IncFIB(AP001918)	APEC/UPEC/ ExPEC

MLST: multilocus sequence typing, cgMLST: core genome MLST, UPEC: Uropathogenic E. coli, ExPEC: extraintestinal E. coli, DAEC:, EAEC: , APEC

Bold: High risk sequence types

Supplementary Table S3: Genetic diversity and virulence profile of *Klebsiella* species recovered from animal, human and environmental sources in Nigeria

ID No	Species	MLST	Serotype	Virulence genes	Plasmids
H2	*K. pneumoniae*	ST501	KL8:O1v2	*iutA*	IncFII(K), Col440I, IncFIB(pKPHS1), IncFIB(K), IncR
H4	*K. pneumoniae*	**ST45**	KL24:O2v1	*fyuA*, *iutA*	IncFIB, IncFII
H5	*K. pneumoniae*	**ST45**	KL52:OL101	*Irp2, iutA*	IncQ, IncFII(K), IncR
H6	*K. pneumoniae*	**ST11**	KL38:O3b	*iutA, traT*	Col440I, IncR, IncFIB(K), INcFII(K)
H7	*K. pneumoniae*	ST36	KL102:O2v2	*iutA*	IncFIA(HI1), Col(MGD2), IncFIB(K)
H22	*K. quasipneumoniae*	ST3266	KL169:OL104	*iutA*, *traT*	IncFII(K), IncR, IncFII, IncFIB(K), Col440I
H23	*K. pneumoniae*	ST307	KL102:O2v2	*fyuA*, *traT*, *iutA*	InCFII(K), IncFIB(K)
H25	*K. pneumoniae*	ST307	KL102:O2v2	*fyuA*, *traT*, *iutA*	InCFII(K), IncFIB(K)
H26	*K. pneumoniae*	ST307	KL102:O2v2	*fyuA*, *traT*, *iutA*	IncFII(K)
H30	*K. quasipneumoniae*	ND	KL35:O5	*iutA*	-
H31	*K. pneumoniae*	ST200	KL125:O3b	*iutA*	-
H36	*K. pneumoniae*	ST252	KL8:O2v2	*iutA*	IncFII(K), IncFIB(K), IncR, Col440I
H39	*K. pneumoniae*	**ST17**	KL169:O104	*iutA*	IncFII(K), Col440I, IncFIB(K), IncFIA(HI1)
H40	*K. pneumoniae*	**ST340**	KL59:O2v1	*traT*, *iutA*	IncR, IncFIB(K), IncFIA(HI1)
H45	*K. pneumoniae*	**ST340**	KL15:O4	*traT*, *iutA*	IncFII(K), Col440I, IncR, IncFIB(K), IncFIA(HI1)
H46	*K. pneumoniae*	**ST340**	KL15:O4	*traT*, *iutA*	IncFII(K), Col440I, IncR, IncFIB(K), IncFIA(HI1), Col3M
H47	*K. pneumoniae*	**ST340**	KL15:O4	*traT*, *iutA*	IncFII(K), Col440I, IncR, IncFIB(K), IncFIA(HI1)
H48	*K. pneumoniae*	ST3271	KL136:O1v2	*traT*, *iutA*	IncFII(K), Col440II, IncR, IncFIB(K), ColRNAI
H50	*K. quasipneumoniae*	ST925	KL159:O101	*iutA*	Col440I
L18	*K. pneumoniae*	ST627	KL54:O1v2	*iutA*, *terC*	IncFIB(Mar), IncHI1B

Bold: High risk sequence types
